# Computational identification of co-evolving multi-gene modules in microbial biosynthetic gene clusters

**DOI:** 10.1038/s42003-019-0333-6

**Published:** 2019-02-28

**Authors:** Francesco Del Carratore, Konrad Zych, Matthew Cummings, Eriko Takano, Marnix H. Medema, Rainer Breitling

**Affiliations:** 1 Manchester Centre for Synthetic Biology of Fine and Speciality Chemicals (SYNBIOCHEM), Manchester Institute of Biotechnology, Faculty of Science and Engineering, University of Manchester, 131 Princess Street, Manchester, M1 7DN United Kingdom; 2 Structural & Computational Biology Unit, European Molecular Biology Laboratory, Meyerhofstraße 1, 69117 Heidelberg, Germany; 3 Bioinformatics Group, Wageningen University, Droevendaalsesteeg 1, 6708PB Wageningen, The Netherlands

## Abstract

The biosynthetic machinery responsible for the production of bacterial specialised metabolites is encoded by physically clustered group of genes called biosynthetic gene clusters (BGCs). The experimental characterisation of numerous BGCs has led to the elucidation of subclusters of genes within BGCs, jointly responsible for the same biosynthetic function in different genetic contexts. We developed an unsupervised statistical method able to successfully detect a large number of modules (putative functional subclusters) within an extensive set of predicted BGCs in a systematic and automated manner. Multiple already known subclusters were confirmed by our method, proving its efficiency and sensitivity. In addition, the resulting large collection of newly defined modules provides new insights into the prevalence and putative biosynthetic role of these modular genetic entities. The automated and unbiased identification of hundreds of co-evolving group of genes is an essential breakthrough for the discovery and biosynthetic engineering of high-value compounds.

## Abstract (web summary)

Francesco Del Carratore et al. present an unsupervised statistical method to detect bacterial biosynthetic gene clusters. They confirm many known modules and identify a large collection of new modules, which may prove useful in the biosynthesis of high-value compounds.

## Introduction

Microbial specialised metabolism is a rich source of high-value and biochemically active compounds of immense biotechnological and biomedical potential^1,2^. The enzymatic pathways responsible for the biosynthesis of such compounds are encoded by physically clustered groups of genes called biosynthetic gene clusters (BGCs). These sometimes very large^3^ genomic regions have a high modular structure at the genetic level^4,5^. For instance, it has been previously observed that certain classes of BGC share co-evolving multi-gene subclusters, which work together as a unit for the implementation of a specific biosynthetic function^4,5^. Numerous examples of such genetic entities have been described^6–19^. BGCs often harbour more than one subcluster, and can be composed almost exclusively of these genetic building blocks, as is the case for aminocoumarins: the groups of genes responsible for the biosynthesis of the deoxysugar ring moiety, the aminocoumarin core and the pyrrole ring moieties each form a discrete subcluster^20,21^. These subclusters are not constrained to the aminocoumarins, however: the pyrrole ring subcluster used in the biosynthesis of at least four different end compounds^20–25^. All these subclusters have been detected through the experimental characterisation of numerous BGCs and provide a very useful benchmark when developing a method able to automatically detect similar genetic entities. The deconstruction of BGCs into subclusters encoding discrete chemical moieties has been used to generate novel compounds by combinatorial biosynthesis^26^; therefore, their discovery and characterisation is potentially a great help to synthetic biology approaches to BGC reconstruction de novo, providing an extremely useful tool for the biotechnology research community aiming at exploiting the full commercial and clinical potential of microbial metabolism. The relevance and the (evolutionary) exchange of these modules across microbial species are still under study, however^4^. Recent advances in computational biology have allowed the identification of millions of putative BGCs^27^ by the systematic analysis of DNA sequence^28^ using, e.g., freely available BGC-mining tools, such as antiSMASH^29^, BAGEL^30^, PRISM^31^, and ClusterFinder^4^. Here, we take this approach one step further: with the intent of elucidating the relevance and exchange of biosynthetic subclusters in the evolution of BGCs, we developed a statistical method for the detection of subclusters. This algorithm successfully detected 185,718 statistically significant putative subclusters (hereafter, called modules) in 12,842 predicted BGCs in microbial sequences from GenBank in a systematic and automated manner. Although accurately detecting already known subclusters, our method is able to rigorously define numerous novel modules and provide new insights into the prevalence of putative functional subclusters and their role in specialised metabolite biosynthesis. The resulting library of statistically defined modules is a rich resource for the specialised metabolite research community. In fact, these modules could be used for the design of BGCs that are likely to encode the biosynthesis of molecules with novel combinations of known chemical moieties. Moreover, these results yield an unprecedented insight into the degree of modular organisation of the specialised biosynthetic machinery across the entire microbial kingdom, and significantly reduce the role played by serendipity in the initial identification of individual modules by guiding the selection of statistically supported promising candidates through a comprehensive and unbiased automated approach. In addition, the method described here is able to identify strongly supported modules with currently unknown functions. Such modules are potentially responsible for the biosynthesis of novel chemical (sub)structures and represent valuable guides for the targeted mining of microbial genomes for new drug candidates. This work will also be a great asset for cluster prediction tools such as antiSMASH^29^, as it can be used to annotate predicted BGCs by suggesting which genes in a BGC function together as discrete units in a complex biosynthetic pathway; in addition, this module-based annotation can also help in cluster boundary prediction.

## Results

### Module detection algorithm

**Fig. 1:**
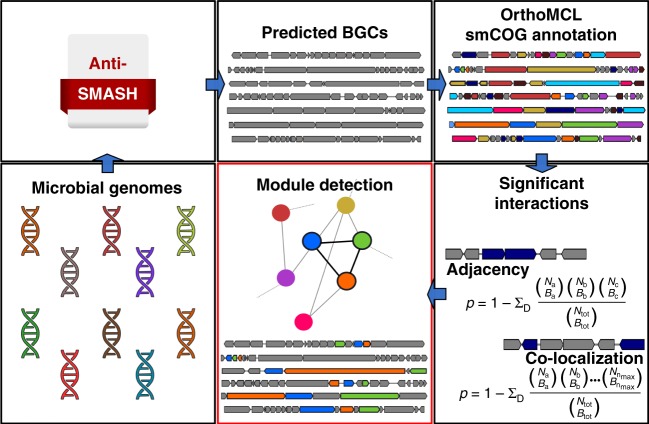
Module detection algorithm. By processing the entire collection of microbial genomes considered in this study, antiSMASH predicted tens of thousands BGCs. OrthoMCL was then used for the smCOG annotation. Next, these smCOGs were organised into a network where two smCOGs are connected only if they share a significant number of adjacency or colocalization interactions. Fully connected sub-graphs (cliques) are considered as putative biosynthetic modules

After generating a collection of predicted BGCs through antiSMASH^29^, the method relied on the orthoMCL package v 1.4^32^ for the annotation of specialised metabolite Clusters of Orthologous Genes (smCOG). All the detected smCOGs were next organised into a network where two smCOG are connected if they share a statistically significant number of adjacency or colocalization interactions (the evaluation of the statistical significance of the number of interactions is described in the Methods). Finally, all the fully connected sub-graphs (i.e., cliques) found in the network were considered as putative biosynthetic modules, this is a statistically very conservative approach, as it requires that all individual interactions between module members to be highly significant. This algorithm successfully detected 185,718 statistically significant putative subclusters (hereafter, called modules) in 12,842 predicted BGCs in microbial sequences from GenBank in a systematic and automated manner. Although this is a rather large number when compared with the number of BGCs present in our data set, it is important to consider that the numbers appear somewhat inflated by the common appearance of groups of nested modules, where smaller (less specific, but statistically highly significant) modules are contained within larger (more specific) modules in various combinations; examples of this biologically important (and expected) phenomenon are discussed below. More importantly, this statistically strongly supported subset is only a tiny fraction of the > 10^34^ possible modules. The method is briefly summarised in Fig. 1, whereas a more detailed description is available in the Methods. Given the large number of statistically significant putative modules detected by our algorithm, a method for ranking the 185,718 putative modules would strongly benefit the exploration of our database. This has been achieved through the Module Interest Benchmarking (MIB) score. After ranking the modules according to different metrics (number of BGCs containing the module, number of BGCs containing the module and present in the MIBiG database, module size, strictest *p*-value threshold, number of different compound classes and their Shannon entropy, and the percentage of smCOGs members of a specific category), the MIB score is simply computed as a weighted sum of all ranks obtained for each module. Subsequently, the obtained values are rescaled over the range from 1 (least interesting) to 100 (more interesting). The default values of the weights used are: 2 for the length of the modules (longer, and thus more specific, being better), 15 for the Shannon entropy, 10 for the number of BGCs containing the module, 5 for the maximum *p*-value threshold, 10 for the percentage of tailoring smCOGs, and 0 for all the remaining criteria. However, all the weights can be adjusted by the user to increase or decrease the contribution of each criterion in the prioritisation. A more detailed description of the prioritisation procedure can be found in the Methods. In order to evaluate the biological and evolutionary relevance of the detected modules, we first checked for the appearance of well-characterised and previously reported modules in our database, starting with the previously described aminocoumarin subclusters^20,21^.

### Aminocoumarin, deoxysugar, and pyrrole ring

**Fig. 2:**
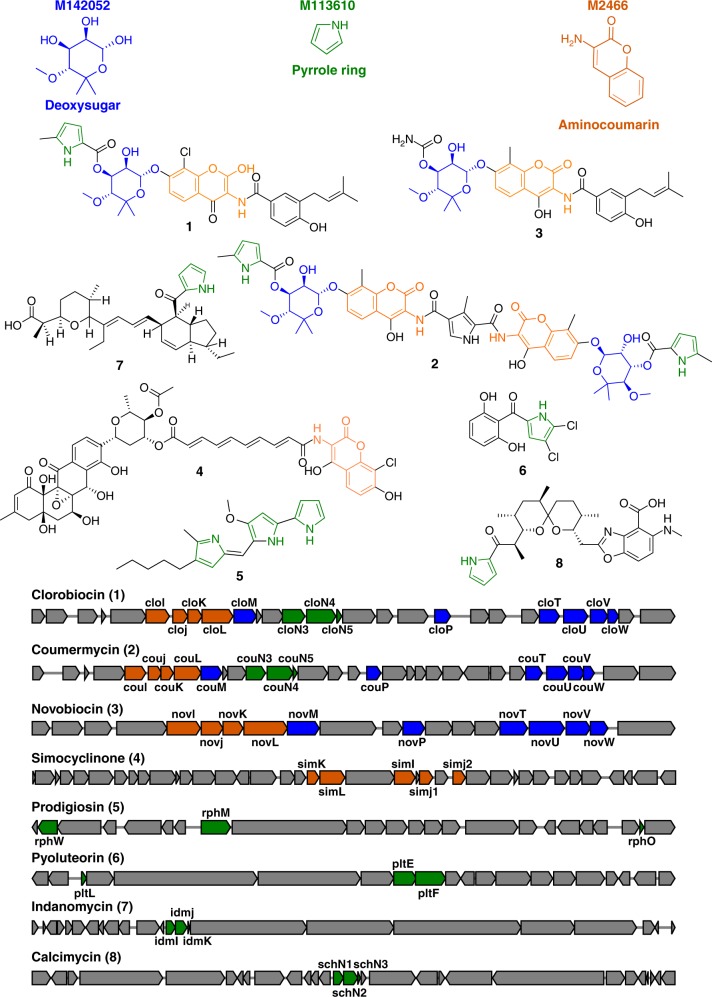
Deoxysugar, pyrrole ring, and aminocoumarin modules. Overview of the clorobiocin, coumermycin, novobiocin, simocyclinone, prodigiosin, pyluteorin, indanomycin, and calcimycin BGCs. When present, the genes covered by Module M142052 (blue), Module M113610 (green), and Module M2466 (orange) are highlighted in the clusters. The chemical moieties related to the modules are highlighted in the chemical structures. Clusters are not drawn to scale

Canonical aminocoumarin-specialised metabolites are highly modular in structure and comprise an aminocoumarin core moiety, which is often decorated with deoxysugars and pyrrole rings (Fig. 2). Each of these distinct chemical moieties is encoded by a discrete suite of genes, a subcluster, which appear to mix-and-match in Nature to produce chemically diverse end compounds. Previously, experimentally validated subclusters encoding the biosynthesis of all three chemical moieties were detected by our bioinformatics analysis. Figure 2 shows 3 modules that perfectly cover three well-known subclusters that are all found both in the clorobiocin and the coumermycin BGCs. Specifically, module M142052 (MIB score = 60.22, number of BGCs covered = 4) covers the group of genes responsible of the biosynthesis the deoxysugar ring present in clorobiocin (*cloM, P, T, U, V, W*), novobiocin (*novM, P, T, U, V, W*), and coumermycin (*couM, P, T, U, V, W*)^20^. Module M2466 (MIB score = 68.32, BGCs = 6) targets the genes encoding the aminocoumarin group in the clorobiocin BGC (*cloI-L*) novobiocin (*novI-L*) and simocyclinone (*simI, J1, J2, K, L*)^20,21^. Module M113610 (MIB score = 95.36, BGCs = 33) covers the three-gene subcluster responsible for the biosynthesis of the pyrrole ring in a number of different BGCs: clorobiocin (*cloN3-N5*)^20^, prodigiosin (*rphW, M, O*)^22^, coumermycin (*couN3-N5*)^20^, calcimycin (*schN1-N3*)^23^, indanomycin (*idmI- K*)^24^, and pyoluteorin (*pltE-L*)^25^.

### ACP-linked PKS extender modules (hierarchy of modularity)

**Fig. 3:**
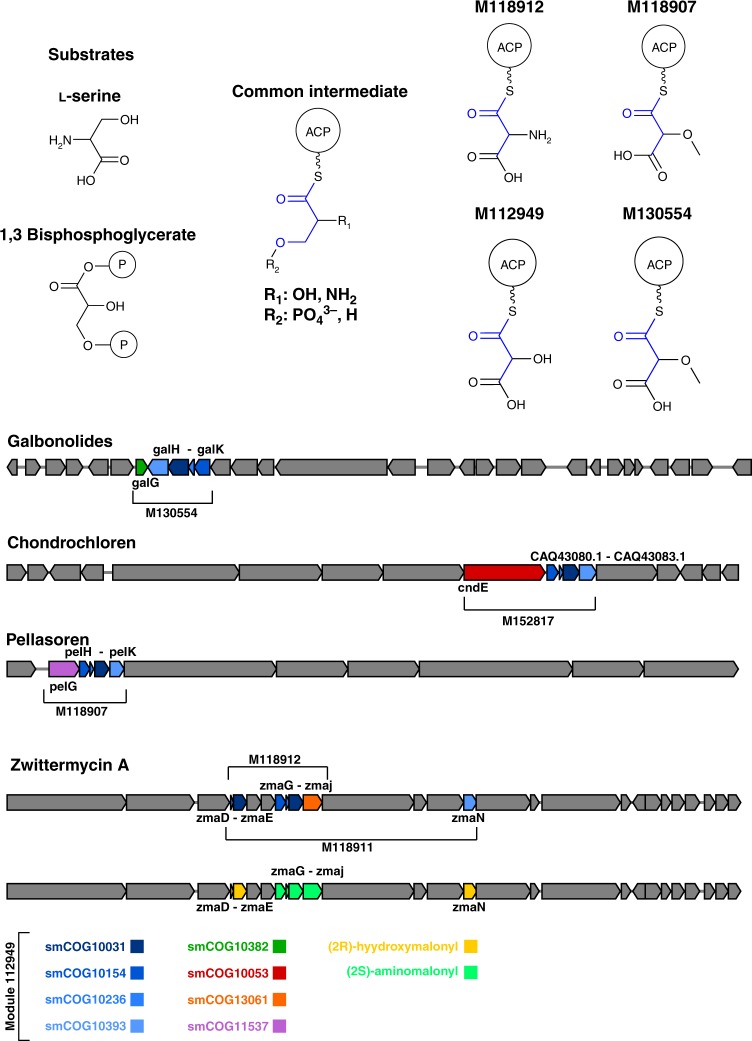
Schematic representation of the chemical moieties produced and the hierarchical organisation of the ACP-linked PKS extender modules. l-Serine and 1,3-biphosphoglycerate are the two possible substrates accepted by the modules. The galbonolides cluster has been chosen as a representative example of the known clusters containing the methoxymalonyl-ACP module. It is also noteworthy that all modules share a common intermediate

The previously described five-gene subcluster responsible for the formation of methoxymalonyl-ACP, another classical example of a functional pathway module, is covered by module M130554 (MIB score = 96.62; containing smCOG10382, smCOG10393, smCOG10031, smCOG10236, and smCOG10154). This module is found in a total of 28 BGCs, 12 of which are fully characterised BGCs found in the MiBiG database^33^: galbonolides^6^, tautomycin^7,8^, oxazolomycin^9,10^, FK520^11,12^, macbecin^13,14^, ansatomycin^13,15^, geldamycin^13,16^, herbimycin^13,16^, concanamycin^17^, bafilomycin^18^, apoptolidin^19^, and nocathiacin^34^. Interestingly, the group of genes *noc-5–noc-9* from the nocathiacin BGC are unlikely to play a role in the biosynthesis of this ribosomally synthesised and post translationally modified peptide. Instead, it is more likely that, in this genetic context, module M130554 forms part of an additional, flanking BGC within the corresponding genome (which is yet to be completely sequenced). Module M130554 encompasses a smaller related module, M112949. This reduced module lacks the *O*-methyltransferase (O-MT) responsible for methylation of the *α*-carbon hydroxyl group (smCOG10382) and is found in a total of 73 BGCs, 15 of which have been fully characterised, and shows a higher MIB score than module M130554 (99.09). Interestingly, module M112949 appears to comprise a minimal complement of genes from which an array of unusual acyl-ACP extender units are derived, e.g., aminomalonyl-ACP (zwittermycin A)^35^, alternative routes to methoxymalonyl-ACP (chondrochloren)^36^ and an unusual glycolate containing-ACP substrate (pellasoren)^37^. In the case of the chondrochloren BGC, the module M112949 cooperates in the synthesis of methoxymalonyl-ACP despite the lack of a discrete gene encoding an O-MT, instead the O-MT function complemented by an enzymatic domain within the polyketide synthase subunit *cndE*^36^ (smCOG10053). The *cndE* O-MT is not part of the module as defined here, as *cndE* is not annotated as a methyltransferase. This indicates how the statistically defined modules can be used for a targeted search of missing enzymes, redundancy within BGCs and functional gene homologues: the observed O-MT domain fusion (smCOG10053) is common, however, and forms part of its own module (M152817, MIB score = 92.83) comprising module M112949 with the addition of smCOG10053, highlighting two convergent routes to methoxymalonyl-ACP formation. The combinatorial complexities of acyl-ACPs do not stop here, however. Module M112949 covers 4 out of the 5 genes responsible for the biosynthesis of a glycolate type extender unit in the pellasoren BGC^37^. All five genes are covered by module M118907, which contains an additional acetyltransferase, ACP and O-MT multifunctional gene product (*PelG*, smCOG11537) predicted to be responsible for the loading, tethering, and methylation of 1,3 bisphosphoglycerate^37^. Furthermore, the zwittermycin A BGC contains a nine-gene subcluster encoding the biosynthesis of two different acyl-ACP PKS extender units: (2S)-aminomalonyl-ACP and (2R)-hydroxymalonyl-ACP^38^. This big subcluster is completely covered by M118911 (MIB score = 87.47), which is composed of module M112949 plus smCOG13061 (*zmaJ*). Deconstruction of this subcluster shows module M112949 to comprise two ACP genes (*zmaD* and *zmaH*, smCOG10236) and two acyl-CoA dehydrogenase genes (*zmaE* and *zmaI*, smCOG10031) which are orthogonal for (2R)-hydroxymalonyl- and (2S)-aminomalonyl-ACP formation correspondingly (Fig. 3, yellow and green ORFs respectively), a single smCOG10154 acyl-ACP dehydrogenase predicted to be promiscous for both acyl-intermediates, and a glyceryl-s-*ZmaD* synthase (smCOG10393), *zmaN*, specific for (2R)-hydroxymalonyl-ACP formation. The biosynthesis of the (2S)-aminomalonyl-ACP necessitates a dedicated seryl-AMP synthetase, *zmaJ*, to load l-serine onto *ZmaH*. This enzymatic domain is annotated by an alternative smCOG, therefore falling outside of the module M112949, instead being completely covered by module M118912 (MIB score = 92.61), composed of smCOG10031, smCOG10154, smCOG10236, and smCOG13061, and eluding to a common small subcluster comprising smCOG10031, smCOG10154, and smCOG10236 (M176566, MIB score = 99.06, BGCs = 99). Figure 3 shows this representative example of the hierarchical organisation of subclusters. Several additional known subclusters have been identified by our method. For example, the biosynthesis of 4-methyl-3-hydroxyanthranilic acid has been associated to module M108999 (Supplementary Fig. 1), whereas the biosynthesis of 2,3-dihydroxy-benzoic acid (DBHA) has been associated to module M21869 (Supplementary Fig. 2). Moreover, the two overlapping modules M103444 and M131293 have been associated to the biosynthesis of 9- and 10-membered enediyne rings (Supplementary Fig. 3). Finally, module M107196 and module M11279 have been associated with the biosynthesis of *β*-carotene and ectoine, respectively (Supplementary Note 1).

### Exploring the full collection of detected modules

As previously mentioned, the MIB score allows us to prioritise the detected modules by considering different criteria with different weights at the same time. Supplementary Data 1 show a selection of the available metrics for the modules previously discussed. It is noteworthy that these modules show a significantly high MIB score, demonstrating that experimentally verified modules are strongly favoured by this prioritisation method. All the previously mentioned modules are in the top 25% when considering their MIB scores. According to the Fisher’s exact test, the probability of observing this situation simply by chance is equal to *p*-value = 5.809 × 10^−10^. This increases our confidence that previously unreported modules with comparably high MIB scores are also biochemically interesting evolutionary units. When selecting the examples for the discussion below, we considered only modules with at least one hit in the MIBiG database^33^, thus focusing on examples where at least some chemical information is available to validate our interpretation, and we iteratively filtered out all the modules containing at least one smCOG found in the modules previously discussed (so that at each step we only consider modules that are clearly different from modules already examined) we then selected the module showing the highest MIB score. With the first iteration we identified the four-smCOG module M63477 (MIB score = 100), composed of smCOG10029, smCOG10228, smCOG10252, and smCOG10310. This fairly common module (found in 77 different BGCs) is found in the streptomycin BGC of *Streptomyces griseus* covering four genes: *argD* SG7F10.54, *argB* SG7F10.53, *argC* SG7F10.51, and *argJ* SG7F10.52. These four genes are encoding the enzymes involved in the biosynthesis of l-ornithine from l-glutamate, and are therefore undoubtedly a functionally and evolutionarily coherent unit. In the case of the streptomycin gene cluster, however, Module M63477 is most a likely part of a neighbouring biosynthetic gene cluster, merged as a result of the greedy nature of the antiSMASH detection algorithm. The wide distribution of this module suggests that the ornithine precursor is more widely used than previously appreciated, possibly as a precursor to diaminopropionate biosynthesis (from ornithine and serine), as has been suggested for the formation of stenothricin^39^.

The second iteration selected the module M100203 (MIB score = 99.55, BGCs = 47), which contains three smCOGs: smCOG10555, smCOG10642, and smCOG11025. Two of the BGCs targeted by this module are present in the MIBiG database, encoding the biosynthesis of A-503083 A and A-500359 A, respectively. In both of these clusters, the module covers three genes that are predicted to encode three subunits of a functional carbon monoxide dehydrogenase complex with an unclear role in biosynthesis^40,41^. The inclusion of this three-gene module across a wide variety of biosynthetic pathways (Shannon’s entropy = 2.26) pinpoints these poorly characterised genes to either play a fundamental, and not yet understood, role in specialised metabolism, or alternatively to result from greedy BGC prediction in a similar fashion to module M63477. In either case this group of genes deserves closer experimental evaluation.

The third iteration highlighted the module M80260 (MIB score = 99.47) as an interesting candidate. This module contains smCOG10066, smCOG10118, and smCOG10495 and is found in 58 BGCs. This module targets three genes in three fully characterised modules: R1128 (*zhuF, D, E*), polyketomycin (*pokAC1, AC2, AC3*), and xantholipin (*xanB3, B1, B2*). This three-gene module appears to be encoding the biosynthesis of malonyl-CoA^14,42,43^. Surprisingly, this module has a rather high Shannon entropy (1.96), and not all of the BGCs are predicted to be involved in polyketide biosynthesis. For example, the module is found in clusters putatively responsible for the biosynthesis of non-ribosomal peptides, terpenes, ectoines, and bacteriocins.

Module M105700 (MIB score = 99.01, BGCs = 47) is the next module selected with this procedure. This module targets only one BGC present in the MIBiG database, which encodes the biosynthesis of the polyketide tetronasin^44^. The module contains three smCOGs: smCOG10139 (covering the tsn3 gene), smCOG10264 (*tsn1/tsn2*), and smCOG10631 (*tsn4*), which encode enzymes similar to the components of pyruvate dehydrogenase and related multi-enzyme complexes; whether these have a role in boosting the supply of acetyl-CoA precursors for specialised metabolite biosynthesis has not been demonstrated, but seems plausible.

The next module selected by our procedure is module M156303 (MIB score = 98.67, BGCs = 31). Interestingly, this novel three-smCOG module (smCOG10062, smCOG10123, and smCOG10687) is found in 15 clusters present in the MIBiG database: landomycin (*lanT, Q, S*)^45^, granaticin (*gra-orf23, orf26, orf27*)^46^, sch47554/sch47555 (*schS1, S3, S2*)^47^, rubradirin (*rubN3, K, L, N4*)^48,49^, spinosad (*spnN, R, Q, O*)^50^, lactonamycin (*lct44, 45, 46*)^51^, kijanimicin (*kijD10, D2, D7, D1*)^52^, tetrocarcin A (ACB37729.1, ACB37732.1, ACB37737.1 and ACB37754.1)^53^, polyketomycin (*pokS4, S5, S3*)^42,54^, streptolydigin (*slgS4, S6, S3*)^55^, pristinamycin (*cpp28, hpaA, cpp32*)^56^, nocathiacin (*nocS4, S6, S5*)^34^, BE-7585A (*bexQ, T, V*)^57^, and amicetin (*amiD, N, C*)^58^. In all cases, this module appears to be involved in sugar modification/biosynthesis. The biochemical details of their function have not been fully elucidated in any of these cases, but the modularity analysis will facilitate a comparative approach to understanding their action. Module M156303 is just one example of a large number of sugar-related modules of high statistical support, highlighting the pervasive modularity of sugar decorations in specialised metabolite biosynthesis, which could be the target of a more detailed evolutionary and biochemical evaluation in the future.

Finally, our procedure selected module M134122 (MIB score = 97.33, BGCs = 20) for further discussion. This module, which is composed of smCOG10818, smCOG11294, smCOG12468, and smCOG12628, targets two BGCs present in the MIBiG database: kanamycin (BAE95427.1, BAE95426.1, BAE95429.1, and BAE95428.1)^59,60^ and tallysomycin (*orf35–37*)^61^. The genes covered by module M134122 code for the subunits of nitrate reductase (*α*, *β*, *γ,* and *δ* chain) and have no known role in the biosynthesis of specialised metabolites. They represent a true (functional) module, but play their role in primary, rather than specialised metabolism. Nevertheless, the fact that this module is found with such high statistical support in the close neighbourhood of many BGCs might provide relevant information about the genetic context in which these BGCs are situated, and potentially the physiological context in which they are activated^62^. Importantly, this example also illustrates that our module detection method could be of more general value beyond specialised metabolism.

The first six modules prioritised using our iterative procedure are summarised in Supplementary Data 2. It should be emphasised that the biological roles suggested for these modules are purely based on plausibility arguments, but experimental validation will be required in each case to establish their precise functional relevance. Exploring the whole collection of putative functional biosynthetic modules detected by the method presented here will take a community effort and is beyond the aims of this work. The modules described above illustrate the power of the statistical detection and definition of putative biosynthetic modules, and the database provided will be a helpful resource for the whole community to explore further.

## Discussion

In this work, we present a statistical method to automatically detect putative functional (and evolutionary) modules in BGCs. The fact that the method is unsupervised makes it powerful in associating genes that may have not been associated before by looking at individual gene clusters. For the future, a key next step will entail correcting for phylogenetic bias in the input data (i.e., having many similar gene clusters from closely related genomes), which currently can lead to the detection of artificial modules (although the Shannon entropy score (see Methods) can be used to down-rank such artefacts). This could be done either by performing additional redundancy filtering on the input data, or by correcting for phylogenetic structure in the statistical tests (which would of course increase the computational demands even more). Nonetheless, the obtained library of automatically predicted modules allows the efficient definition of BGCs that are likely to encode the biosynthesis of molecules with novel combinations of known chemical moieties. Moreover, strongly supported modules with currently unknown functions can be identified in our data, which potentially are responsible for the biosynthesis of discrete and novel chemical (sub)structures. In addition, the whole collection of automatically detected modules will help understand the degree of modularity in the organisation of microbial BGCs, and it will provide useful tools to be used in conjunction with other screening modalities for drug discovery by genome mining. For example, this work will also be a great asset for cluster prediction tools such as antiSMASH^29^, as it can be used to annotate BGCs by predicting, which genes in a BGC function together as discrete units in a complex biosynthetic pathway, i.e., the enediynes (see Supplementary Note 1), and can also help in cluster boundary prediction. In the future, we intend to integrate the module library with public web services such as antiSMASH^29^, antiSMASH database^63,64^, and MIBiG^33^. In this context, it is noteworthy that the MIBiG database already allows users to upload chemically characterised subclusters.

## Methods

### Data acquisition

All the available bacterial and fungal genomic sequences were obtained from GenBank (access date 08 October 2012). We used antiSMASH version 1.0^65^ to detect all BGCs in this set of genomes, resulting in a collection of 482,040 genes in 14,869 BGCs.

### Cluster trimming

The boundaries of BGCs reported by antiSMASH^65^ are expanded to include genes neighbouring the actual biosynthetic cluster in order to assure that the complete genomic entity is extracted. This greedy approach is a rational choice for molecular biology purposes, but would result in extra computational burden in our downstream analysis. To minimise this problem, we analysed the data set with ClusterFinder^4^ to obtain the most probable cluster borders, based on each cluster’s constituent PFAM domains. We shortlisted PFAM domains that are important for specialised metabolites (Supplementary Data 3) and trimmed genes from the extremes of each of the clusters if their probability of having a PFAM domain from the list was lower than 0.1. The threshold value was selected based on the observation that increasing the threshold up to 0.1 resulted in more genes being trimmed out, whereas any further increase (up to 0.5) had hardly any effect. Trimming removed 135,298 genes from the set. The trimmed data set consisted of 346,742 genes in 14,809 BGCs.

### Clusters of orthologous genes

A set of smCOGs was constructed from all genes in the set of BGCs using the orthoMCL v 1.4 package^32^ with standard settings. OrthoMCL analysis resulted in 19,292 smCOGs. From this set, we removed smCOGs having fewer than three genes, resulting in 12,756 smCOGs. We also removed 45,906 genes that did not belong to any of the remaining smCOGs. This left us with 211 BGCs that were empty (i.e., did not include any gene belonging to any smCOG), further narrowing the data set down to 300,612 genes in 14,598 BGCs. In order to reduce redundancy, if two or more clusters showed the same smCOG composition (regardless the order), we only kept the shortest one, narrowing the data set down to 12,842 non-redundant BGCs. Subsequently, we annotated the smCOGs based on the annotation of the genes they contain. For this purpose, we divided the annotations of individual genes into five major categories: core biosynthesis, regulator, tailoring, transport, and other. This taxonomy is described in more detail in Supplementary Data 4. Each smCOG was annotated: (1) if > 60% of genes from a smCOG share the same annotation category, this category was used a main annotation of the smCOG with exception of (2) if the most common category is other but the second most frequent one occurs in > 40% of genes, the second category was used; (3) if the most common category is present in < 60% of genes but first and second most common categories are together present in > 75% of the genes the smCOG was annotated with a double category (e.g., tailoring/core); (4) otherwise, the smCOG was annotated as mixed. Exact descriptions of all smCOG annotations are available in the Supplementary Data 5.

### Interactions between smCOGs

A putative module is defined as a set of smCOGs of any size found in more BGCs than expected by chance. Considering all the 12,842 different smCOGs present in our data set, the number of possible modules is enormous, even if the size of a module is constrained to be between three and ten genes:

1 $$\mathop {\sum}\limits_{n = 3}^{10} \left(\begin{array}{c}12842\\ n\end{array}\right) \cong 3.35 \times 10^{34}$$

Handling such an enormous number of potential modules represents a very complex computational challenge. To address this issue, we focused on the pairwise interactions between different smCOGs, i.e., the detection of smCOGs that co-occur surprisingly often within the same BGCs. In practice, we distinguished between two types of interactions: adjacency, when two smCOGs appear side-by-side in a BGC, and colocalization, when they are found together within the same cluster independently of their relative position. Adjacency and colocalization interactions were counted for each possible pair of smCOGs. Genes in the BGCs that did not belong to any of the smCOGs did not contribute to the number of interactions, but their positions were not skipped; i.e., a smCOG neighbouring two genes that did not belong to any smCOG would be counted as having no adjacency interactions in this cluster.

### Assessment of statistical significance of interactions

Considering two smCOGs sharing *N* adjacency or colocalization interactions, one can assess the statistical significance of such interactions by computing the probability of observing at least *N* interactions between the two COGs, if they were randomly distributed among the clusters present in our data set. Although easy to state, this calculation is far from trivial computationally, and tackling it with a permutation-based approach would be too computationally demanding. However, for both kinds of interactions, it is relatively easy to compute the probability of observing at least *N* interactions, keeping fixed the positions of one of the two smCOGs in our data set. Using this approach, it is possible to obtain two (different) *p-*values per each pair of smCOGs, depending on which of them is considered as fixed. Aiming for the most conservative approach, we considered only the larger of the two *p*-values when determining the statistical significance of an smCOG interaction in the subsequent analysis.

### Adjacency interactions

Consider a pair of smCOGs (called COG A and COG B) that occur adjacently at least once. If we keep all genes annotated as belonging to COG A in their original positions, one could divide all the remaining positions in the BGCs into three classes: (a) positions that are not adjacent to any COG A gene; (b) positions adjacent to one COG A gene; and (c) positions adjacent to two COG A genes. *N*_*a*_, *N*_*b*_, and *N*_*c*_ represent the number of available positions in each of these three classes. The total number of adjacency interactions between the two COGs is:

2 $$i_{orig} = B_b + 2 \cdot B_c$$

where *B*_*b*_ represent the number of COG B genes occupying a position adjacent with one COG A genes and *B*_*c*_ represent the number of COG B gene occupying a position adjacent with two COG A genes. The number of COG B genes occupying a position not adjacent to any COG A gene is indicated as *B*_*a*_. It should be noticed that the same number of interactions can be observed with more than one distribution of the COG B. The probability of observing one specific distribution *d* (i.e., a specific set of values for *B*_*a*_, *B*_*b*_, and *B*_*c*_) when the COG A genes are fixed and the COG B genes are randomly distributed among all the available positions is expressed by the following hypergeometric equation:

3 $$P_D = \frac{{ \left(\begin{array}{c}N_{a}\\ B_{b}\end{array}\right) \left(\begin{array}{c}N_{b}\\ B_{b}\end{array}\right)\left(\begin{array}{c}N_{c}\\ B_{c}\end{array}\right)}}{{\left(\begin{array}{c}N_{tot}\\ B_{tot}\end{array}\right)}}$$

where *B*_*tot*_ and *N*_*tot*_ represent the total number of COG B genes present in the data set and the total number of available positions. The probability of observing at least *i*_*orig*_ adjacency interactions (i.e., the *p*-value) can be easily computed as:

4 $$p = P_{i \ge i_{orig}} = 1 - P_{i < i_{orig}} = 1 - \mathop {\sum}\limits_{\mathbf{D}} P_d$$

where **D** represent the set of all the possible combinations of *B*_*a*_, *B*_*b*_, and *B*_*c*_, leading to a number of interactions lower than the observed one.

### Colocalization interactions

The calculations for the colocalization interactions are analogous to those described for the adjacency interactions. However, the number of gene classes is not limited to three, but to the maximum number of occurrences of COG A genes in a single cluster (*n*_*max*_):

5 $$P_D = \frac{{\left(\begin{array}{c}N_a\\ B_a\end{array}\right)\left(\begin{array}{c}N_b\\ B_b\end{array}\right)\cdots\left(\begin{array}{c}N_{n_{max}}\\ B_{n_{max}}\end{array}\right)}}{{\left(\begin{array}{c}N_{tot}\\ B_{tot}\end{array}\right)}}$$

This creates a computational problem whenever *n*_*max*_ is large. In order to avoid this problem, we removed such redundant smCOGs for the colocalization calculations: when genes from the same smCOG are found more than once in a cluster, they are substituted by an empty position and the genes from the affected smCOG are attached at the end of the cluster separated by an empty position from the rest of the cluster. Althoughthis approach deletes some adjacency interactions and slightly changes the topology of some clusters, it leads to conservative *p*-value estimates and, most importantly, makes the *p*-value computations easier, as equation 5 simplifies to:

6 $$P_D = \frac{{\left(\begin{array}{c}N_a\\ B_a\end{array}\right)\left(\begin{array}{c}N_b\\ B_b\end{array}\right)}}{{\left(\begin{array}{c}N_{tot}\\ N_{tot}\end{array}\right)}}$$

All the *p*-values computed for both kinds of interactions were corrected for multiple testing using the Benjamini–Yekutieli method^66^ for controlling the false-discovery rate under dependency.

### Module detection

The obtained multiple-testing corrected *p*-values were used for detecting putative modules. By selecting an initial arbitrary *p*-value threshold for significant interactions, it is possible to compute a binary matrix M of dimension (*C* × *C*), where *C* is the total number of smCOGs present in our data set, and the *m*_*i*,*j*_ element of the matrix is equal to 1 if either the adjacency or the colocalization *p*-value is lower or equal to the chosen threshold. This matrix represents an undirected graph, where two smCOGs (nodes) are connected by an edge if they share a statistically significant number of adjacency or colocalization interactions. All the maximal cliques found in this graph and containing at least three elements are detected and added to our list of putative modules. A maximal clique is a fully connected sub-graph, where connections are based on either significant adjacency or significant colocalization, which is not a subset of any other fully connected sub-graph. All *p*-values occurring in the data set smaller or equal to 0.1 are iteratively considered as the arbitrary *p*-value threshold. The graph analysis and the maximal clique detection was performed using the *igraph* package^67^. Using this approach, we ended up with a total of 197,564 putative modules. It is important to remember that each three-member module is supported by three individually significant interactions, and that depending on the intended use case, stricter *p*-value thresholds can easily be applied to reduce the number of modules for further analysis. Although a rigorous estimation of the false discovery rate associated with our module detection method is not provided, we considered all the modules found together in less than two BGCs as false positives. Such modules represent ~ 6% of all the detected modules and they have been removed from our database.

### Modules prioritisation and trimming

A number of different metrics were computed for each detected module in order to prioritise and filter them according to user-defined criteria:

- Number of BGCs containing the module. As the modules are defined on the basis of individual pairwise interactions between smCOGs, it is possible that all pairwise interactions between members of a module are significant, even when the complete module never occurs together in the same BGC. To remove such spurious modules, we removed ~ 6% of the detected modules that were found together in less than two of the BGCs, resulting on a total of 185,718 modules.
- Number of BGCs containing the module and present in the MIBiG data set. The MIBiG data set of experimentally characterised BGCs^33^ was queried in order to identify which of the BGCs present in our data set have been associated with experimental data. For these clusters, the enzymatic pathway is at least partially defined and the chemical structures of the end compound known.
- Module size. The size of the detected modules (i.e., the number of smCOGs) ranges from 3 (minimum value allowed by the module detection algorithm) to a maximum of 42 (the largest maximal clique using the most lenient interaction threshold). In total, 80% of modules are smaller than 16 smCOGs, the median size is 9, and the most likely value is 3. Very large modules are in fact typically entire BGCs, rather than biosynthetic modules (subclusters), and are therefore usually not of interest for the subsequent analysis (although they obviously are modules in the sense of being coherent evolutionary entities, the detection of which confirms the validity of the module detection algorithm).
- Strictest *p*-value threshold. While detecting the modules, a different *p*-value threshold is chosen at each iteration. For each module, it is possible to identify the strictest *p*-value threshold that can be used to detect it. This value can be considered as a measure of the overall statistical significance of the module.
- Number of different compound classes and their Shannon entropy. While looking for putative BGCs, antiSMASH is also predicting the chemical class of the end compound. Using this information, we can focus specifically on modules that occur in clusters responsible for the most diverse set of predicted compound classes—these are the most likely to be responsible for carrying out well-defined chemical functions and to act as independent evolutionary units. To accurately estimate the diversity of compound classes covered by the BGCs containing a specific module, we used Shannon’s informational Entropy (SE), which is computed as follows:

7 $$\mathop {\sum}\limits_{i = 1}^I f_i \cdot {\mathrm{log}}(f_i)$$

where *f*_*i*_ is the ratio between the number of times the module is involved in the biosynthesis of the *i*^th^ compound class and the total number of BGCs containing the module, and *I* is the total number of different putative end compound classes produced by these BGCs. The higher the SE, the larger is the number of targeted compound classes, and the lower the bias toward one or more compound classes.

- Percentage of smCOGs members of a specific category. As mentioned above, all smCOGs were annotated according to functional categories (e.g., transport, tailoring, core biosynthesis etc.). For each module, we computed the percentage of each functional category. Using this information, we can, for example, focus on modules composed only or mostly of tailoring smCOGs (again, these are most likely to represent evolutionary units of interest).

For the overall prioritisation, we used a weighted combination of all of these metrics, the MIB score. This score is simply computed as a weighted sum of all the ranks obtained considering each metric individually. Subsequently, the MIB scores obtained are rescaled over the range from 1 (least interesting) to 100 (most interesting). Depending on the user particular interest, the weights can be adjusted to increase the contribution of individual criteria to the overall prioritisation. The default values of the weights used in the subsequent discussion are: 2 for the length of the module (longer, and thus more specific, being better), 15 for the Shannon entropy, 10 for the number of BGCs containing the module, 5 for the maximum *p*-value threshold, 10 for the percentage of tailoring smCOGs, and 0 for all remaining criteria.

### Code availability

The code used for the *p*-value evaluation, module detection, and metrics computation was written in R^68^ with the use of the *igraph*^67^ and *Rmpfr*^69^ packages, and it is available at https://github.com/francescodc87/Modules_Detection together with a detailed documentation.

### Reporting summary

Further information on experimental design is available in the Nature Research Reporting Summary linked to this article.

## Supplementary information

Supplementary Information

Supplementary data 1

Supplementary data 2

Supplementary data 3

Supplementary data 4

Supplementary data 5

Description of Supplementary Data

Reporting Summary

## Data Availability

The complete data set is available at https://github.com/francescodc87/Modules-explorer together with a *Shiny*-based^70^ web application, which provides users with a simple graphical interface to explore the data set containing all the detected modules. A detailed documentation is present the github pages. In addition, all the supplementary material mentioned in the manuscript can be also found at https://github.com/francescodc87/Modules_Detection/tree/master/Supplemetary_Files.
