# Supplementary material for: Computational identification of co-evolving multi-gene modules in microbial biosynthetic gene clusters
Source: Commun Biol. 2019 Feb 28;2:83. doi: 10.1038/s42003-019-0333-6 (PMC6395733; doi:10.1038/s42003-019-0333-6)
Supplement: Supplementary file 1 — Supplementary Information [file 42003_2019_333_MOESM1_ESM.pdf]

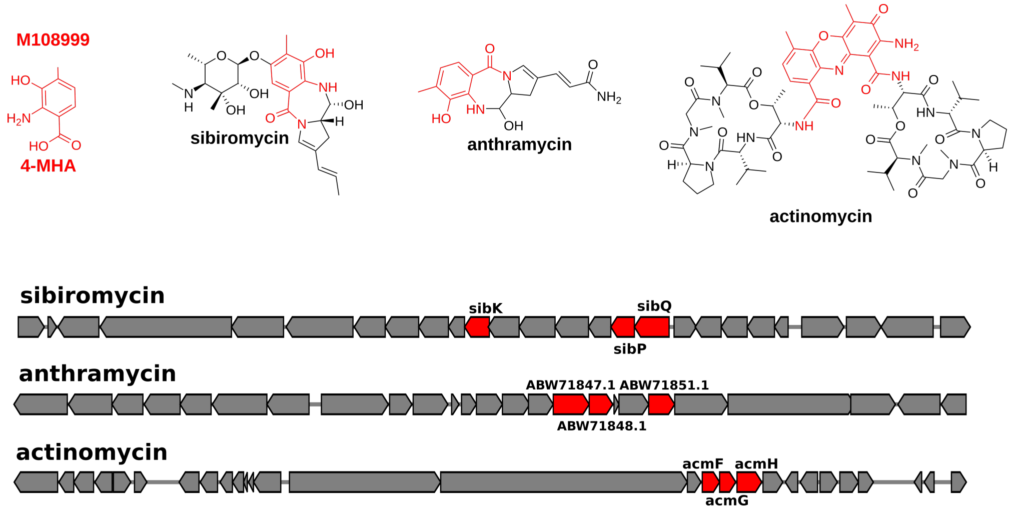

Supplementary Figure 1: 4-methyl-3-hydroxyanthranilic acid (4-MHA). The biosynthesis of 4-methyl-3-hydroxyanthranilic acid (4-MHA) is encoded by a sub-cluster of 3 genes present in at least 3 different BGCs: sibiromycin (*sibK, P, Q*) [1], actinomycin (*acmF, G, H*)[2] and anthramycin (ABW71851.1, ABW71848.1 and ABW71847.1)[3]. As shown in Supplementary Figure 1, this sub-cluster corresponds to module M108999 (containing smCOG10870, smCOG11911 and smCOG12486; MIB score $= 87.54$, found in 11 BGCs). Furthermore, the module is present in the antimycin BGC. In the antimycin biosynthetic pathway, homologs of these same three genes are involved in the biosynthesis of 3-aminosalicylate[4], which is structurally closely related to the final precursor of 4-MHA, 3-hydroxyanthranilicacid (3-HA). The methyltransferase that converts 3-HA to 4-MHA (which is not part of module M108999 because it is only present in a minority of the BGCs) is missing in the actinomycin pathway, which explains the additional structural difference. Interestingly, the shared genetic basis of the biosynthesis of these two nonproteinogenic amino acids had not been noted in literature thus far. Additionally, module M108999 contains seven uncharacterized BGCs from diverse organisms, including *Deinococcus*, *Streptomyces*, *Streptosporangium*, and *Nocardia* species. The 4-MHA-type sub-clusters in these BGCs are associated with a wide variety of core scaffold biosynthesis genes, including NRPSs, type I PKSs, type III PKSs and terpene cyclases. This suggests that biosynthetic pathways have evolved in which 4-MHA-like moieties are incorporated into a wide range of scaffolds. The detection of this cluster as statistically significant, despite the fact that the genes involved are not always contiguous, shows the power of the co-occurrence analysis included in the module detection algorithm. The 4-MHA moiety highlighted in the structures.

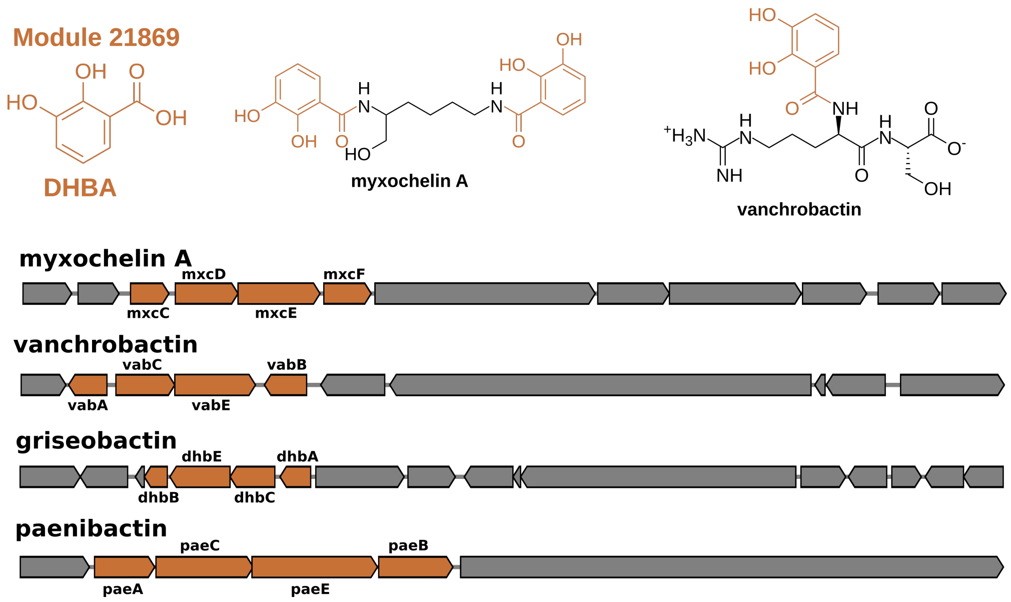

Supplementary Figure 2: 2,3-dihydroxy-benzoic acid (DBHA). Another well-known sub-cluster is represented by a group of 4 genes encoding the biosynthesis of 2,3-dihydroxy-benzoic acid (DHBA)[5]. This sub-cluster has been described in several characterised BGCs: myxochelin A (*mxcC, D, E, F*)[6], vanchrobactin (vabA, C, E, B)[7], paenibactin (*paeA, C, E, B*)[8], griseobactin (*dhbA, C, E, B*)[9], enterobactin (*entA, C, E, B*)[10] and vibriobactin (*vibA, C, E, B*)[11]. As shown in Supplementary Figure 2, module M21869 perfectly covers this sub-cluster for 4 of the above mentioned clusters (MIB score = 77.58, number of BGCs covered = 299). Whilst the vibriobactin cluster is not present in our initial dataset, module M21869 does not target the BGC encoding enterobactin. This BGC is targeted instead by module M21893 (MIB score = 58.94, BGCs covered = 306), comprising a subset of module M21869, lacking the smCOG covering the *entA* gene, as *entA* is assigned to a different smCOG (smCOG11931, often annotated as amino acid adenylation domain) by our automated annotation algorithm. This case illustrates two main points: (1) the use of variable significance thresholds allows the detection of partial matches to biosynthetic modules, and (2) the module detection algorithm is robust towards minor misannotations in individual gene clusters, which will be unavoidable for any large-scale genomic analysis. The DHBA moiety highlighted in the structures.The structures for griseobactin and paenibactin are not available in ChemSpider nor PubChem.

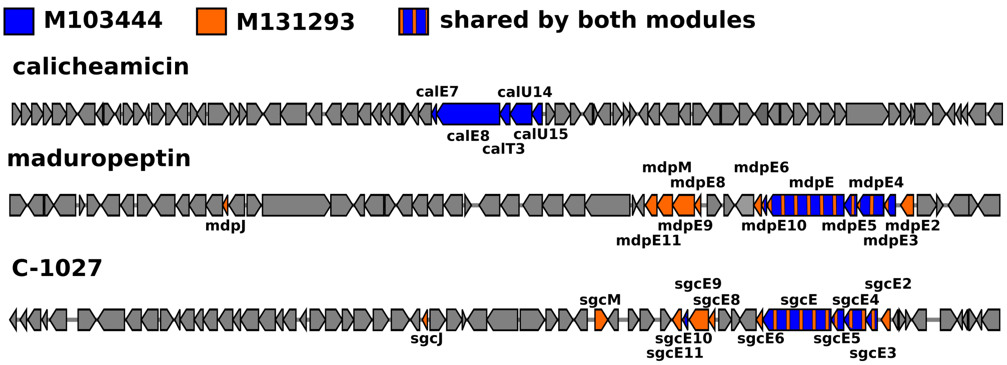

Supplementary Figure 3: Enedyines. The biosynthetic logic of enedyine specialised metabolites remains largely enigmatic when compared to other polyketides[12]. Irrespectively, our analysis identified one module, M103444, to completely cover the group of genes responsible for the core biosynthesis of both 9- and 10-membered enedyines[13] indiscriminately. The existence of a sub-cluster of core genes conserved between 9 and 10-membered enediyne BGCs is well reported in literature, and this gene cassette is routinely used as a probe to mine sequence data for new and exotic enediyne-specialised metabolites[14, 15]. The factors discriminating between the biosynthesis of 9- and 10-membered enediyne rings remain unknown however, but are thought to occur beyond the "core" biosynthetic genes within module M103444. Genome neighbourhood network analysis of 10 characterised enediyne BGCs highlighted 9 genes to associate specifically with biosynthesis of 9-membered enediynes (D2, E2, E8, E9, E11, F, J, L and M), and 4 genes to associate with 10-membered enediyne ring biosynthesis (R3, S6, T5 and U20)[14], none of which are covered by module M103444. Interestingly, as shown in Figure 3, module M103444 is encompassed within a larger module, M131293, which appears to be specific to 9-membered enediynes and in addition to module M103444, comprise 6 of the 9 9-member specific genes (E2, E8, E9, E11, J and M). The genes covered by module M03444 (blue), module M131293 (orange) or both (blue-and-orange stripes) are highlighted.

Supplementary Note 1 - Ectoine and β-carotene

Module M107196 (MIB score $= 83.44$) contains 3 smCOGs (smCOG1005, smCOG10036 and smCOG10195) that cover genes involved in the biosynthesis of β-carotene from geranylgeranyl diphosphate (GGPP)[16,17]. This module is found in 139 BGCs, 8 of which have been experimentally characterised and reported using the MIBiG standard[18]. Despite the wide distribution of this module among the predicted BGCs present in our dataset, it is always found in very similar genetic contexts; therefore, it shows a very low Shannon entropy value (0.47). In fact 94% of the BGCs are annotated as terpenes, and all the others are annotated as hybrid clusters also including terpenes (e.g. terpene-bacteriocin). All 8 chemically characterised BGCs produce highly similar carotenoids where the "sub-cluster" is responsible for biosynthesis of the core specialised metabolite, opposed to a discrete chemical moiety. A similar situation is found with module M112779 (MIB score $= 82.40$), which encodes the enzymatic machinery necessary for ectoine biosynthesis[19]. This small module containing 3 smCOGs (smCOG10060, smCOG10107 and smCOG10163, corresponding to *ectB*, *ectC* and *ectA*), is prevalent, being found in 235 BGCs, and has a very low Shannon entropy value (0.37). Such low Shannon entropy indicates that these modules exist as discrete BGCs encoding ectoine and carotenoids, rather than a module within a larger parent BGC synthesising a complex hybrid specialised metabolite, e.g. simocyclinone, calicheamicin or coumermycin.

1. Li, W., Khullar, A., Chou, S., Sacramo, A. & Gerratana, B. Biosynthesis of sibiromycin, a potent antitumor antibiotic. *Applied and Environmental Microbiology* **75**, 2869–2878 (2009).

2. Katz, E. & Weissbach, H. Biosynthesis of the actinomycin chromophore; enzymatic conversion of 4-methyl-3-hydroxyanthranilic acid to actinocin. *Journal of Biological Chemistry* **237**, 882–886 (1962).

3. Hurley, L. H. Elucidation and formulation of novel biosynthetic pathways leading to the pyrrolo[1, 4]benzodiazepine antibiotics anthramycin, tomaymycin, and sibiromycin. *Accounts of Chemical Research* **13**, 263–269 (1980).

4. Schoenian, I. *et al.* An unprecedented 1, 2-shift in the biosynthesis of the 3-aminosalicylate moiety of antimycins. *ChemBioChem* **13**, 769–773 (2012).

5. Fischbach, M. A., Walsh, C. T. & Clardy, J. The evolution of gene collectives: How natural selection drives chemical innovation. *Proceedings of the National Academy of Sciences* **105**, 4601–4608 (2008).

6. Li, Y., Weissman, K. J. & Müller, R. Myxochelin biosynthesis: direct evidence for two-and four-electron reduction of a carrier protein-bound thioester. *Journal of the American Chemical Society* **130**, 7554–7555 (2008).

7. Balado, M., Osorio, C. R. & Lemos, M. L. A gene cluster involved in the biosynthesis of vanchrobactin, a chromosome-encoded siderophore produced by *Vibrio anguillarum*. *Microbiology* **152**, 3517–3528 (2006).

8. Wen, Y. *et al.* Identification and analysis of the gene cluster involved in biosynthesis of paenibactin, a catecholate siderophore produced by *Paenibacillus elgii* B69. *Environmental Microbiology* **13**, 2726–2737 (2011).

9. Patzer, S. I. & Braun, V. Gene cluster involved in the biosynthesis of griseobactin, a catechol-peptide siderophore of *Streptomyces* sp. ATCC 700974. *Journal of Bacteriology* **192**, 426–435 (2010).

10. Crosa, J. H. Genetics and molecular biology of siderophore-mediated iron transport in bacteria. *Microbiological Reviews* **53**, 517–530 (1989).

11. Wyckoff, E. E., Stoebner, J. A., Reed, K. E. & Payne, S. M. Cloning of a *Vibrio cholerae* vibriobactin gene cluster: identification of genes required for early steps in siderophore biosynthesis. *Journal of Bacteriology* **179**, 7055–7062 (1997).

12. Annaval, T. *et al.* Crystal structure of thioesterase SgcE10 pupporting common polyene intermediates in 9-and 10-membered enediyne core biosynthesis. *ACS Omega* **2**, 5159–5169 (2017).

13. Horsman, G. P., Chen, Y., Thorson, J. S. & Shen, B. Polyketide synthase chemistry does not direct biosynthetic divergence between 9-and 10-membered enediynes. *Proceedings of the National Academy of Sciences* **107**, 11331–11335 (2010).

14. Rudolf, J. D., Yan, X. & Shen, B. Genome neighborhood network reveals insights into enediyne biosynthesis and facilitates prediction and prioritization for discovery. *Journal of Industrial Microbiology & Biotechnology* **43**, 261–276 (2016).

15. Yan, X. *et al.* Strain prioritization and genome mining for enediyne natural products. *mBio* **7**, e02104–16 (2016).

16. Sedkova, N., Tao, L., Rouvière, P. E. & Cheng, Q. Diversity of carotenoid synthesis gene clusters from environmental *Enterobacteriaceae* strains. *Applied and Environmental Microbiology* **71**, 8141–8146 (2005).

17. Lee, J. H. & Kim, Y. T. Cloning and characterization of the astaxanthin biosynthesis gene cluster from the marine bacterium *Paracoccus haeundaensis*. *Gene* **370**, 86–95 (2006).

18. Medema, M. H. *et al.* Minimum information about a biosynthetic gene cluster. *Nature Chemical Biology* **11**, 625–631 (2015).

19. Reshetnikov, A. S., Khmelenina, V. N. & Trotsenko, Y. A. Characterization of the ectoine biosynthesis genes of haloalkalotolerant obligate methanotroph "*Methylomicrobium alcaliphilum* 20Z". *Archives of Microbiology* **184**, 286–297 (2006).
