# Supplementary material for: Computational identification of co-evolving multi-gene modules in microbial biosynthetic gene clusters
Source: Commun Biol. 2019 Feb 28;2:83. doi: 10.1038/s42003-019-0333-6 (PMC6395733; doi:10.1038/s42003-019-0333-6)
Supplement: Supplementary file 7 — Description of Supplementary Data [file 42003_2019_333_MOESM7_ESM.pdf]

Description of Additional Supplementary Files

File Name: Supplementary Data 1
Description: Selection of the available metrics for the modules discussed in the manuscript

File Name: Supplementary Data 2
Description: Selection of the modules prioritized by our approach.

File Name: Supplementary Data 3
Description: List of the PFAM domains that are considered important for specialized metabolites.

File Name: Supplementary Data 4
Description: Annotation of individual genes into five major categories: core biosynthesis, regulator, tailoring, transport, and other.

File Name: Supplementary Data 5
Description: Exact descriptions of all smCOG annotations.
