# Supplementary material for: Computational identification of co-evolving multi-gene modules in microbial biosynthetic gene clusters
Source: Commun Biol. 2019 Feb 28;2:83. doi: 10.1038/s42003-019-0333-6 (PMC6395733; doi:10.1038/s42003-019-0333-6)
Supplement: Supplementary file 8 — Reporting Summary [file 42003_2019_333_MOESM8_ESM.pdf]

nature research

Corresponding author(s): Rainer Breitling

Last updated by author(s): Jan 14, 2019

Reporting Summary

Nature Research wishes to improve the reproducibility of the work that we publish. This form provides structure for consistency and transparency in reporting. For further information on Nature Research policies, see Authors & Referees and the Editorial Policy Checklist.

Statistics

For all statistical analyses, confirm that the following items are present in the figure legend, table legend, main text, or Methods section.

n/a	Confirmed	
☐	☒	The exact sample size (*n*) for each experimental group/condition, given as a discrete number and unit of measurement
☒	☐	A statement on whether measurements were taken from distinct samples or whether the same sample was measured repeatedly
☐	☒	The statistical test(s) used AND whether they are one- or two-sided<br>*Only common tests should be described solely by name; describe more complex techniques in the Methods section.*
☒	☐	A description of all covariates tested
☐	☒	A description of any assumptions or corrections, such as tests of normality and adjustment for multiple comparisons
☒	☐	A full description of the statistical parameters including central tendency (e.g. means) or other basic estimates (e.g. regression coefficient) AND variation (e.g. standard deviation) or associated estimates of uncertainty (e.g. confidence intervals)
☐	☒	For null hypothesis testing, the test statistic (e.g. *F*, *t*, *r*) with confidence intervals, effect sizes, degrees of freedom and *P* value noted<br>*Give P values as exact values whenever suitable.*
☒	☐	For Bayesian analysis, information on the choice of priors and Markov chain Monte Carlo settings
☒	☐	For hierarchical and complex designs, identification of the appropriate level for tests and full reporting of outcomes
☒	☐	Estimates of effect sizes (e.g. Cohen's *d*, Pearson's *r*), indicating how they were calculated

Our web collection on statistics for biologists contains articles on many of the points above.

Software and code

Policy information about availability of computer code

Data collection	All the available bacterial and fungal genomic sequences were obtained from GenBank (access date 08 October 2012). We used antiSMASH version 1.0 to detect all BGCs in this set of genomes, resulting in a collection of 482,040 genes in 14,869 biosynthetic gene clusters.
Data analysis	We analysed the dataset with ClusterFinder to obtain the most probable cluster borders. A set of Specialised Metabolite Clusters of Orthologous Groups (smCOGs) was constructed from all genes in the set of BGCs using the orthoMCL package with standard settings. All remaining analysis relied on custom code, which is described in detail in the manuscript and is available at available at https://github.com/francescodc87/Modules_Detection

For manuscripts utilizing custom algorithms or software that are central to the research but not yet described in published literature, software must be made available to editors/reviewers. We strongly encourage code deposition in a community repository (e.g. GitHub). See the Nature Research guidelines for submitting code & software for further information.

Data

Policy information about availability of data

All manuscripts must include a data availability statement. This statement should provide the following information, where applicable:

- Accession codes, unique identifiers, or web links for publicly available datasets
- A list of figures that have associated raw data
- A description of any restrictions on data availability

The complete dataset is available at https://github.com/francescodc87/Modules-explorer together with a Shiny-based web application which provides users with a simple graphical interface to explore the dataset containing all the detected modules.

Field-specific reporting

Please select the one below that is the best fit for your research. If you are not sure, read the appropriate sections before making your selection.

☐ Life sciences ☐ Behavioural & social sciences ☒ Ecological, evolutionary & environmental sciences

For a reference copy of the document with all sections, see nature.com/documents/nr-reporting-summary-flat.pdf

Ecological, evolutionary & environmental sciences study design

All studies must disclose on these points even when the disclosure is negative.

Study description	Bacterial genome data were analysed to identify co-evolving modules of genes within secondary metabolite biosynthetic gene clusters.
Research sample	All the available bacterial and fungal genomic sequences from GenBank (access date 08 October 2012).
Sampling strategy	All biosynthetic gene clusters detected by antiSMASH were include in the analysis
Data collection	antiSMASH genome annotation
Timing and spatial scale	n.a.
Data exclusions	n.a.
Reproducibility	n.a.
Randomization	n.a.
Blinding	n.a.

Did the study involve field work? ☐ Yes ☒ No

Reporting for specific materials, systems and methods

We require information from authors about some types of materials, experimental systems and methods used in many studies. Here, indicate whether each material, system or method listed is relevant to your study. If you are not sure if a list item applies to your research, read the appropriate section before selecting a response.

Materials & experimental systems

n/a	Involved in the study
☒	☐ Antibodies
☒	☐ Eukaryotic cell lines
☒	☐ Palaeontology
☒	☐ Animals and other organisms
☒	☐ Human research participants
☒	☐ Clinical data

Methods

n/a	Involved in the study
☒	☐ ChIP-seq
☒	☐ Flow cytometry
☒	☐ MRI-based neuroimaging
